# A diselenobis-functionalized magnetic catalyst based on iron oxide/silica nanoparticles suggested for amidation reactions

**DOI:** 10.1038/s41598-022-19030-w

**Published:** 2022-09-01

**Authors:** Reza Taheri-Ledari, Fateme Sadat Qazi, Mahdi Saeidirad, Ali Maleki

**Affiliations:** Catalysts and Organic Synthesis Research Laboratory, Department of Chemistry, Iran University of Science and Technology, 16846-13114 Tehran, Iran

**Keywords:** Chemistry, Materials science, Nanoscience and technology

## Abstract

In this study, a new heterogeneous magnetic catalytic system based on selenium-functionalized iron oxide nanoparticles is presented and suggested for facilitating amide/peptide bonds formation. The prepared nanocatalyst, entitled as “Fe_3_O_4_/SiO_2_-DSBA” (DSBA stands for 2,2′-diselanediylbis benzamide), has been precisely characterized for identifying its physicochemical properties. As the most brilliant point, the catalytic performance of the designed system can be mentioned, where only a small amount of Fe_3_O_4_/SiO_2_-DSBA (0.25 mol%) has resulted in 89% reaction yield, under a mild condition. Also, given high importance of green chemistry, convenient catalyst particles separation from the reaction medium through its paramagnetic property (ca. 30 emu·g^−1^) should be noticed. This particular property provided a substantial opportunity to recover the catalyst particles and successfully reuse them for at least three successive times. Moreover, due to showing other excellences, such as economic benefits and nontoxicity, the presented catalytic system is recommended to be scaled up and exploited in the industrial applications.

## Introduction

Over the time, micro and nanoscale heterogeneous catalytic systems have attracted an increasing attention due to several reasons, such as high-efficiency, convenient separation, well recyclability, biocompatibility, and consistency with the green chemistry’s principles^1–3^. Among various types of the heterogeneous catalysts, the systems based on iron oxide (Fe_3_O_4_) magnetic nanoparticles are very interesting because they are easily synthesized. Moreover, their surfaces can be modified, and they can be separated from the reaction medium by using an external magnet. This easy separation from the reaction medium is an important step towards green chemistry because the requirement of the organic solvents use in the separation and purification processes is completely addressed^4–11^. Surface-coating of the Fe_3_O_4_ nanoparticles with different layers increases the surface area ratio and causes their surface to be tightly functionalized with the desired functional groups^12^.

Organocatalysts, are small organic molecules that can catalyze the synthetic reactions in the absence of the metals or metal ions^13–17^. One of the major challenges of organocatalysts’ utilization is their separation and reusability. The stabilization of these catalysts onto the nanoparticle surfaces, especially Fe_3_O_4_ magnetic nanoparticles, can be an excellent resolution for addressing this challenge^18–20^. In addition to providing a solid support for the organic catalytic sites, utilization of Fe_3_O_4_ includes several other advantages in comparison with the other species. From the chemical aspect, since the surface of the Fe_3_O_4_ nanoparticles is full of hydroxyl functional groups, it would be quite possible to functionalize that with different species through covalent bonding^21,22^. So far, there have been several reports about the composition of the organic compounds with the Fe_3_O_4_ nanoparticles, through which great upshots in various applications were observed^23,24^. From the physical aspect, the structural stability and thermal resistance (and also resistance against oxidation and degradation) is one of the main contributors to the wide utilization of the Fe_3_O_4_ nanoparticles^25^. The mentioned excellence has provided this possibility to recycle these materials and reuse them for several times^26^. Moreover, great paramagnetic property of the Fe_3_O_4_ nanoparticles has led to more convenient separation, which is of high importance in the field of catalysis^27^. Besides, there are biological and environmental justifications (e.g. nontoxicity, biocompatibility and biodegradability) for the use of these materials that are seriously regarded by green chemistry principles^28,29^. However, in this work, we intend to take advantage from those features that are effective in the catalysis scope.

One of the most challenging synthetic reactions in the scope of organic chemistry, is peptide bond formation in the solution phase^30,31^. In this way, various expensive substances such as “TBTU” (2-(1H-benzotriazole-1-yl)-1,1,3,3-tetramethylaminium tetrafluoroborate), “HBTU” 2-(1H-benzotriazole-1-yl)-1,1,3,3-tetramethyluronium hexafluorophosphate), and “HATU” (1-[bis(dimethylamino)methylene]-1H-1,2,3-triazolo[4,5-b]pyridinium 3-oxide hexafluorophosphate) are traditionally used as the *peptide coupling reagent*^32^. So far, various methods have been developed, in which the application of the functionalized nanomaterials (nanocatalysts) has been particularly noticed^33,34^. In the pharmaceutical industry, amide bond formation is one of the most common transformations in medicinal chemistry laboratories^35,36^. Because of proton exchange between the coupling partners, the ideal approach for amide synthesis, i.e., the direct condensation of a carboxylic acid and an amine group with the production of one equivalent of water (dehydration) as the sole byproduct, is not practicable. This interaction can only occur under forcing conditions (such as high temperatures^37^ and microwave irradiation^38^), making it incompatible with the chemical complexity demonstrated by existing therapeutic candidates^39^. This is why, design and manufacture of different types of amide coupling reagents has always been a topic of interest for many researchers around the world for many years.

Among various types of the functionalized nanomaterials, organoselenium compounds such as ebselen^40,41^, diphenyl diselenide^42^, and selenocysteine^43^ are very important and valuable reagents in the organic synthesis due to their high activity^44^. Recently, diselenide bonds have been developed as one type of novel dynamic covalent bonds because of their modulable dynamic behaviors similar to disulfide bonds. Since both elements sulfur and selenium belong to the chalcogen of the periodic table, they exhibit the same chemical properties, so the chemical bonds of diselenide and disulfide show similar behaviors. Compared to the disulfide bonds, the bonding energy of a diselenide bond is lower (diselenide bonds: 172 kJ mol^−1^; disulfide bonds: 240 kJ mol^−1^), so the diselenide bonds are more labile to be dynamically broken and reconstructed than disulfide bonds^45^. So far, there have been several reports about the use of diselenide species as a prone center for chemical catalysis purposes. For example, in 2018, Rangraz et al. using the nano-(Fe_3_O_4_@SiO_2_-Se)_2_, which contained the catalytic site of diselenide (Se-Se), could catalyze the conversion of various aldehydes to their corresponding carboxylic acids with high yield^44,46,47^.

Based on the above information, in this work, an attempt has been made to develop a novel methodology for the amide bond formation by the amino acids, without any need to the conventional coupling reagents. For the first time, a diselenide aromatic structure is loaded onto a heterogeneous supporting substrate, via covalent attachment. The target diselenide compound (DSBA, 2,2′-diselanediylbis benzamide) was initially synthesized, and then covalently attached onto the amine-functionalized Fe_3_O_4_/SiO_2_ nanostructures. As a brilliant point of this thesis, it should be noticed that the prepared diselenide nanocatalyst is recovered under the air atmosphere, and physically recovered and reused through its paramagnetic feature. From mechanistic aspect that have been previously approved by kinetic studies, a reduction/oxidation (red/ox) process is passed through using a small amount of triethyl phosphite^48^. Also, the final dehydration is assisted by the silica network (in the structure of catalyst), which acts as a great molecular sieve^32^. To the best of our knowledge, this is the first time that a magnetic diselenide nanocatalyst (with general formulation of Fe_3_O_4_/SiO_2_-DSBA) is applied in the amide/peptide coupling reactions. High catalytic performance of the proposed system has been clarified by the optimization reactions. Concisely, it was revealed that high reaction yields are obtained in the dipeptide synthesis reactions by using a small amount of the prepared Fe_3_O_4_/SiO_2_-DSBA catalytic system, over a short time (180 min), at room temperature.

## Results and discussion

### Preparation of Fe_3_O_4_/SiO_2_-DSBA catalytic system

#### Synthesis of DSBA organic compound

**Figure 1:**
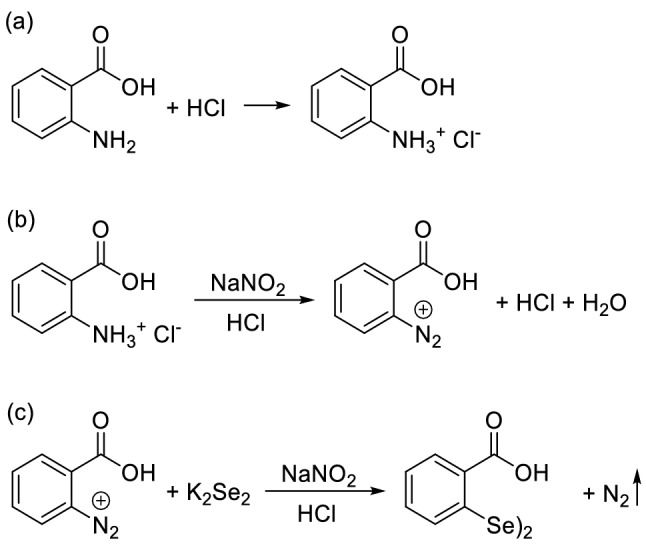
Reactions that take place in different stages of the synthesis of 2,2′-diselanediyldibenzoic acid.

**Figure 2:**
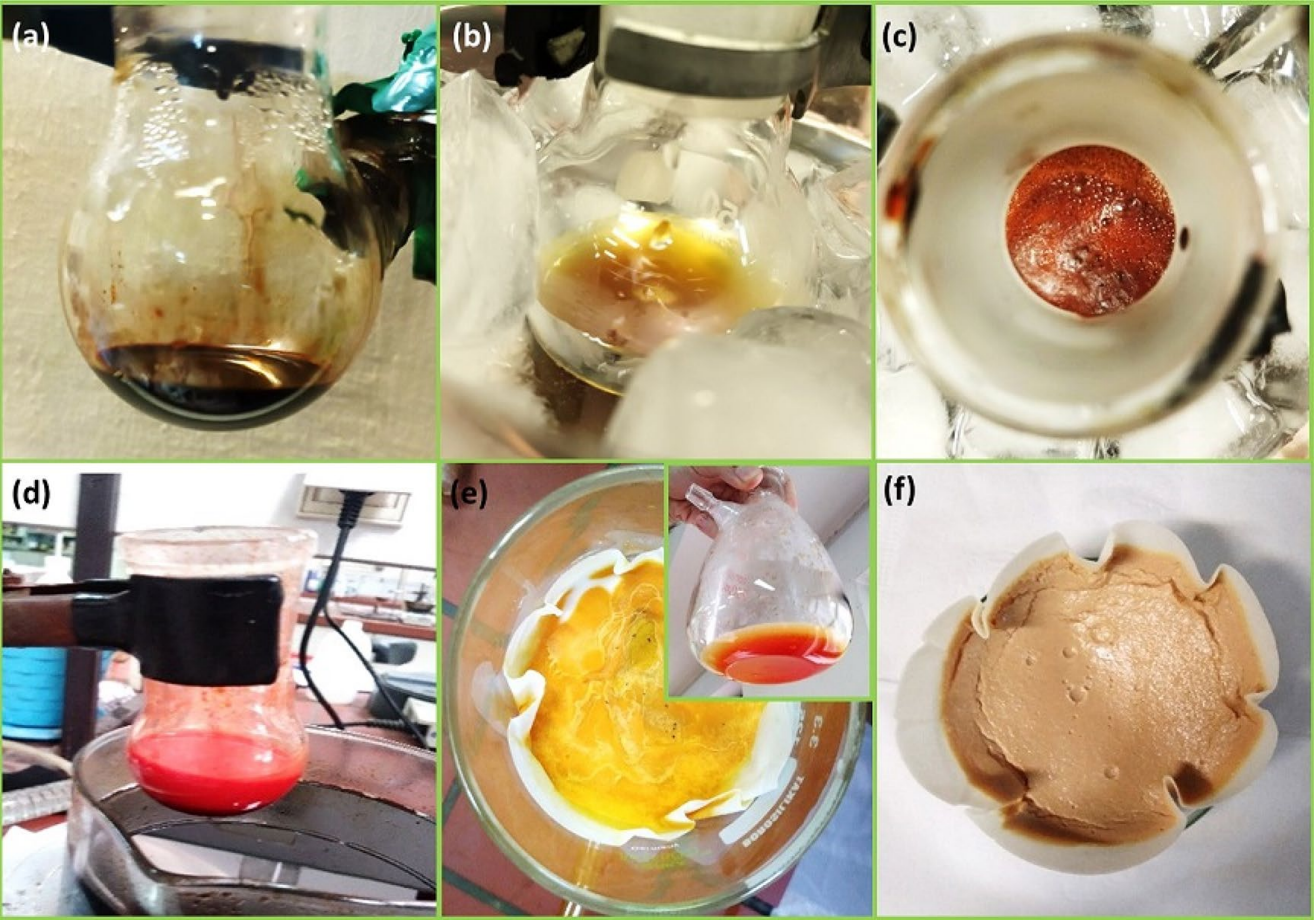
Digital images of: (**a**) synthesis of K_2_Se_2_ metal salt, (**b**) synthesis of 2-carboxybenzenediazonium chloride, (**c**) synthesis of 2,2′-diselanediyldibenzoic acid (N_2_ release creates the bubbles), (**d**) synthesis of 2,2′-diselanediyldibenzoic acid after stirring for 2 h at 90 °C, (**e**) filtered unreacted selenium and oxidized selenium by celite pad, and filtrate including 2,2′-diselanediyldibenzoic acid, and (**f**) 2,2′-diselanediyldibenzoic acid sediment after addition of hydrochloric acid (1 M) (recrystallization has been presented in Video #1).

According to Fig. 1, several steps were required to prepare the Fe_3_O_4_/SiO_2_-DSBA catalytic system. First, the metal salt of potassium diselenide was made using selenium, potassium hydroxide, and potassium borohydride. The important point in the synthesis of this salt was high reactivity with oxygen, which resulted in a very foul-smelling gray substance. Therefore, great care was taken to synthesize this salt under the nitrogen atmosphere. The synthesis of potassium diselenide metal salt was performed simultaneously with the synthesis of 2-carboxybenzenediazonium chloride. To synthesize 2-carboxybenzenediazonium chloride, anthranilic acid was dissolved in the hydrochloric acid solution (Fig. 1a). Simultaneously, NaNO_2_ was dissolved in water and then added to 2-carboxybenzenediazonium chloride solution, and then stirred at zero temperature (Fig. 1b). In this stage, it should be noticed that forming a red color mixture originating from diazonium salt means that the synthesize process is failed. In the next step, the synthesized metal salt of potassium diselenide was added to the solution inside the ice bath, which foamed due to generation of nitrogen gas during the process (Fig. 1c). At the end of this step it was very important to check the pH of the solution. The acidic pH values indicate that there are still some primary reactants in the medium that did not react with the potassium diselenide salt. At this point, by alkalizing the environment, the excess hydrochloric acid of the environment is neutralized leading to a complete consumption of all primary reactants in the environment. Afterward, to eliminate the unreacted selenium and oxidized selenium from the products, the solution was filtered through a thin celite pad. Hydrochloric acid was then added to the filtered solution and then the solid product was filtered through paper filter. In the last step, the resulted sediment was recrystallized in hot methanol to purify the product (see Video #1 Diselenobis Recrystallization)^49^. The appearance of the obtained products from successive stages of the DSBA synthesis process is illustrated in Fig. 2.

#### Preparation of Fe_3_O_4_@SiO_2_-DSBA catalytic system

**Figure 3:**
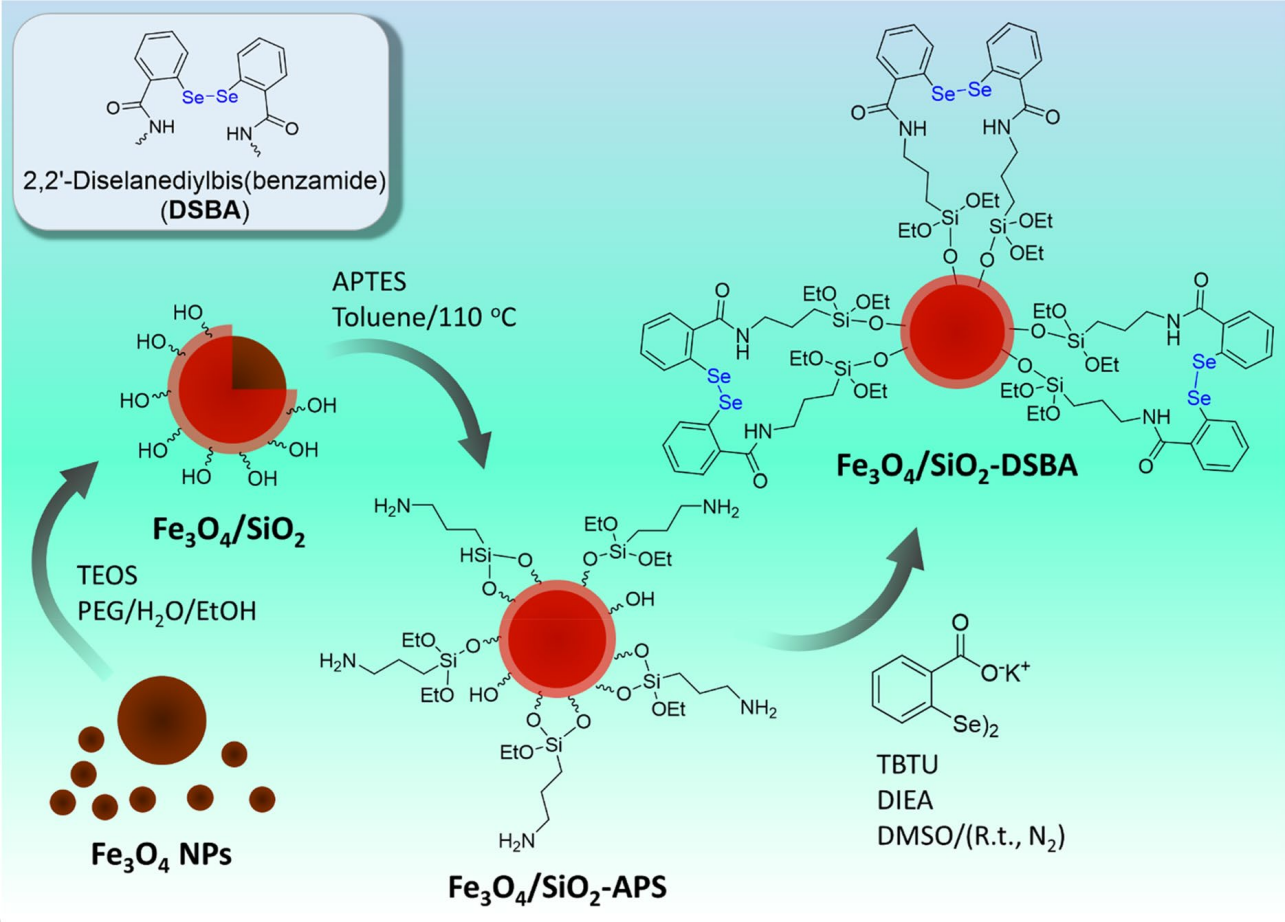
Schematic presentation of the preparation route of Fe_3_O_4_@SiO_2_-DSBA catalytic system.

To turn our nanocatalyst into a heterogeneous magnetic nanocatalyst, as-synthesized 2,2′-diselanediyldibenzoic acid was loaded onto the amine-modified Fe_3_O_4_ magnetic nanoparticles. To synthesize the Fe_3_O_4_ magnetic nanoparticles, iron (II) and iron (III) chloride salts were used under alkaline conditions provided by concentrated ammonium solution^50^. The formed dark precipitations were collected by an external magnet and washed several times with deionized water, ethanol, and acetone. To increase hydroxyl groups onto the surface of magnetic nanoparticles (MNPs), they were coated with a silica (SiO_2_) network using tetraethylorthosilicate (TEOS). Since amine functional groups can form an amide bond with the carboxylic acid functional groups present in the structure of the synthesized catalysts, 3-aminopropyl triethoxysiane (APTES) was used to modify the surface of the Fe_3_O_4_@SiO_2_ nanoparticles^51^. Figure 3 schematically represents the preparation route of the Fe_3_O_4_@SiO_2_-DSBA catalytic system.

### Characterization of Fe_3_O_4_@SiO_2_-DSBA catalytic system

Different equipment and methods were exploited for characterization of the prepared Fe_3_O_4_/SiO_2_-DSBA catalytic system. Fourier-transform infrared (FTIR) spectroscopy was used to examine the functional groups of the new nanocatalyst. Practically, KBr tablets containing the samples were prepared and studied by FTIR spectrometer. Energy-dispersive X-ray (EDX) spectroscopy was used to investigate the presence of different elements in the whole stages of the preparation process. Field-emission scanning-electron microscopy (FESEM) were used to examine the size and morphology of the samples, and electron-transmission microscopy (TEM) was utilized to examine the core–shell structure of the catalyst. To prepare the samples for these imaging methods, the particles were ultrasonicated by a cleaner bath (50 kHz, 100 W L^−1^) for two minutes, at room temperature. Then, dispersions in ethanol were then poured onto the glass laminates. The magnetic properties of the final catalyst were investigated using a vibrational-sample magnetometer (VSM). The thermal resistance and decomposition state of the prepared nanocatalyst was studied in a thermal range of 50–800 °C, by a thermogravimetric analysis (TGA). To ensure that there would not be any probability of oxidation during the TGA study, argon atmosphere was subjected to the sample during the study. X-ray diffraction (XRD) analysis was performed in order to better understand the properties and structure of the catalyst. The brand and model of the used equipment are listed in the experimental section (Table 3).

#### FTIR spectroscopy

The FTIR spectra were used to study the functional groups present in the various compounds produced during the preparation of the Fe_3_O_4_/SiO_2_-DSBA nanocatalyst. According to Figures S1–S4, given in the Supporting Information (SI) section, the peak at ca. 578 cm^−1^ (in all the spectra) is related to the stretching vibration of the Fe–O bond confirming the formation of the iron oxide nanoparticles^52^. In addition, the bands at ca. 803 and 1082 cm^−1^ (Figures S2–S4, in SI section) are attributed to the stretching vibration of Si–O and the asymmetric stretching vibration of Si–O–Si, respectively^53^. In the spectrum of Fe_3_O_4_@SiO_2_-NH_2_ particles (Figure S3), the stretching and bending vibrations of the amine groups have been appeared at ca. 3432 and 1629 cm^−1^, corroborating that aminopropyl silane (APS) has been successfully placed onto the Fe_3_O_4_@SiO_2_ surfaces^54–57^. The formed amide groups in the structure of the Fe_3_O_4_/SiO_2_-DSBA catalytic system verifies the covalent attachment of 2,2′-diselenobis (benzoic acid) (DSBA) onto the surfaces. In this regard, in the spectrum of Fe_3_O_4_/SiO_2_-DSBA (Figure S4), the peaks that appeared at ca. 1629 and 1383 cm^−1^ correspond to C=O and C–N, respectively^58,59^. Also, the peaks related to the stretching vibrations of C–H and C–C bonds present in the aromatic rings seem to be overlapped with the other peaks (Figure S4).

#### EDX analysis

**Figure 4:**
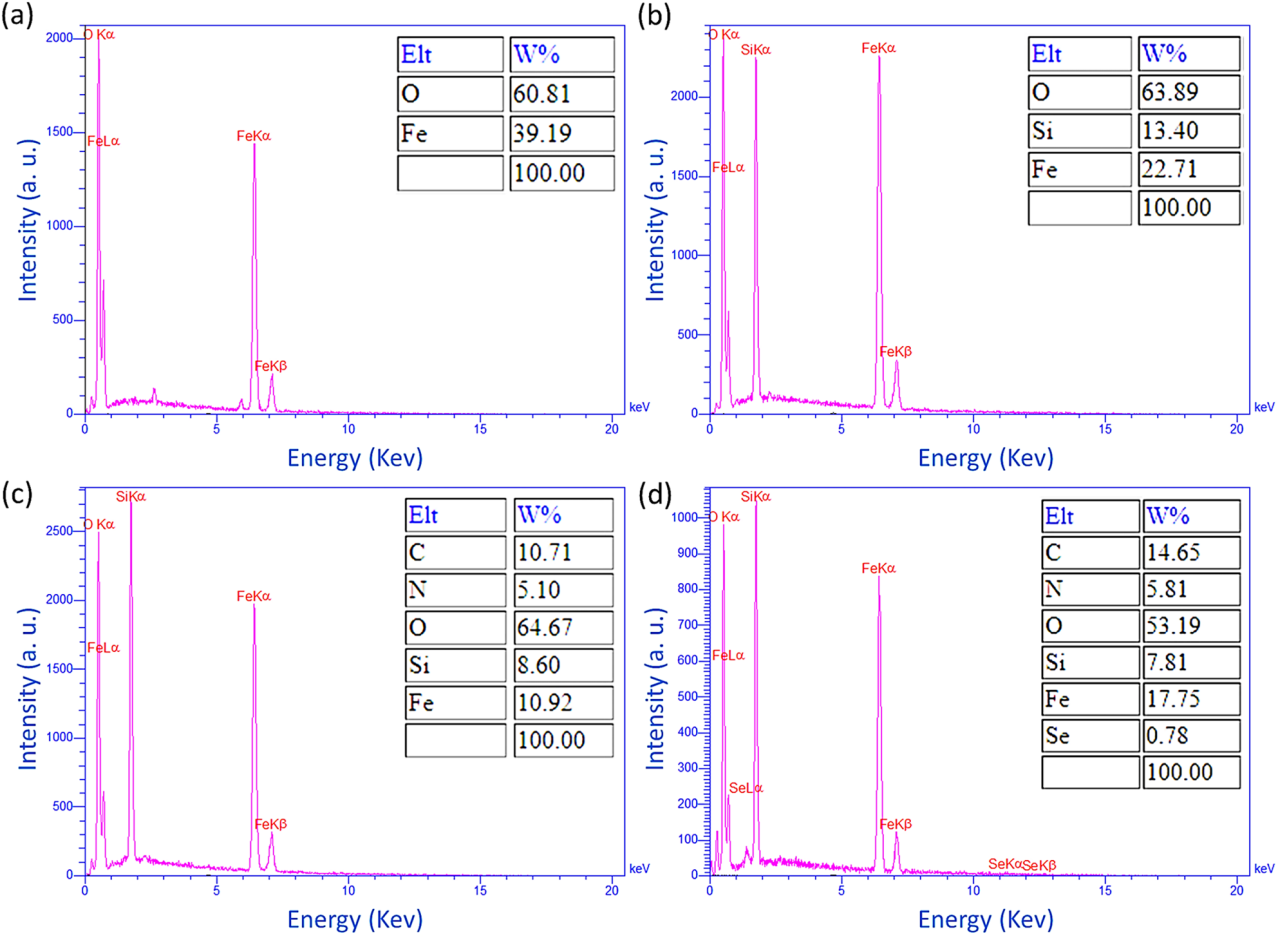
EDX spectra of (**a**) Fe_3_O_4_, (**b**) Fe_3_O_4_@SiO_2_, (**c**) Fe_3_O_4_@SiO_2_-NH_2_, and (**d**) Fe_3_O_4_/SiO_2_-DSBA.

The EDX spectroscopy was utilized to further confirm the existence of elements that are predicted to be present at various stages of nanocatalyst preparation. Figure 4 shows the EDX results of Fe_3_O_4_, Fe_3_O_4_@SiO_2,_ Fe_3_O_4_@SiO_2_@NH_2_, and Fe_3_O_4_/SiO_2_-DSBA nanoparticles. Figure 4a is related to the Fe_3_O_4_ nanoparticles, which expectedly corroborates the presence of Fe and O in the sample. Figure 4b confirms the presence of Fe, O, and Si elements in the structure of Fe_3_O_4_@SiO_2_ nanoparticles. The presence of C and N elements in addition to Fe, O, and Si in Fig. 4c origins from successful modification of the surface of Fe_3_O_4_@SiO_2_ nanoparticles by APS layer. In Fig. 4d, surface attachment of 2,2′-diselenobis benzoic acid onto the Fe_3_O_4_@SiO_2_@NH_2_ particles is verified by the appearance of Se element’s peaks. Also, this is observed that the weight ratio (wt%) of the C element has increased to 14.65% after attachment of 2,2′-diselenobis benzoic acid, well confirming the addition of a new ingredient into the structure.

#### VSM analysis

**Figure 5:**
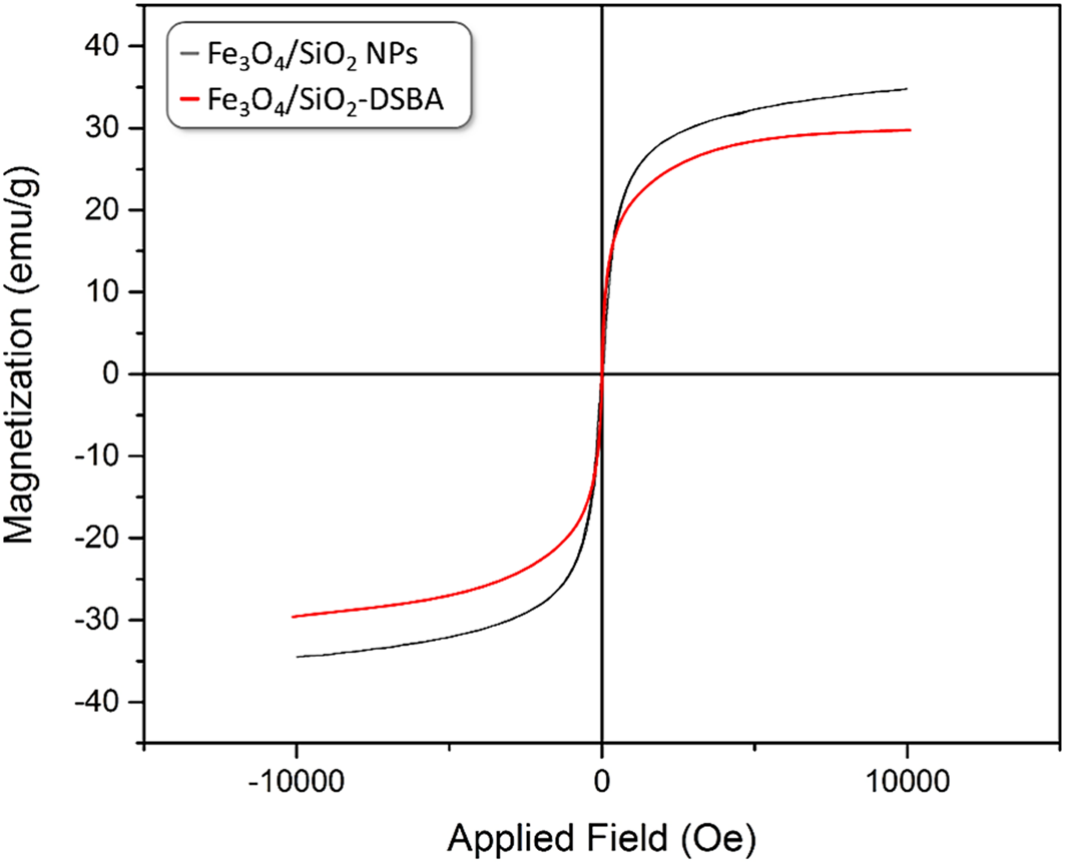
VSM curves of Fe_3_O_4_/SiO_2_-DSBA nanoparticles (red) and Fe_3_O_4_/SiO_2_ nanoparticles (black).

One of the most important features of the prepared catalyst is its easy separation from the reaction mixture by an external magnet. This property of Fe_3_O_4_/SiO_2_-DSBA catalytic system that origins from the presence of Fe_3_O_4_ nanoparticles, has been investigated by vibrating-sample magnetometer (VSM) analysis, as shown in Fig. 5^60,61^. This featured behavior of the Fe_3_O_4_/SiO_2_-DSBA catalytic system is particularly bolded in recyclization process, where the particles can be conveniently separated through holding an external magnet at the bottom of the flask, and reused multiple times. Obviously, the magnetic property of the Fe_3_O_4_ nanoparticles is decreased after formation of a successive layers onto the surfaces. More precisely, the magnetic property of Fe_3_O_4_/SiO_2_ nanoparticles is ca. 35 emu·g^−1^, while this value reduced to ca. 30 emu·g^−1^ after conversion to Fe_3_O_4_/SiO_2_-DSBA structure. However, this amount of magnetization has demonstrated to be quite adequate for execution of the magnetic separation during the catalytic process.

#### XRD analysis

**Figure 6:**
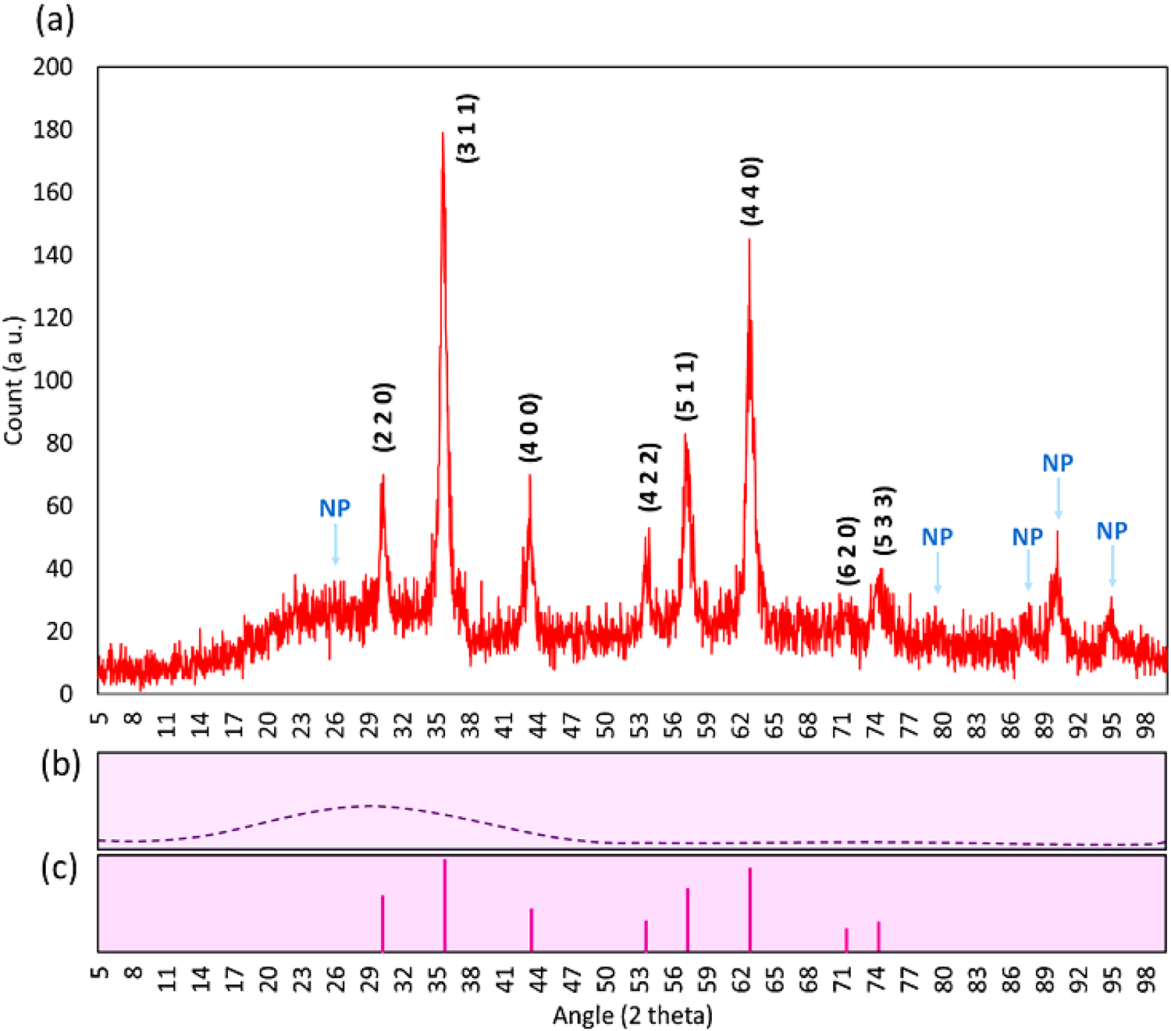
XRD pattern of: (**a**) Fe_3_O_4_/SiO_2_-DSBA catalytic system, (**b**) SiO_2_ NPs, and (**c**) Fe_3_O_4_ NPs. *NP: new peaks*, are attributed to the new crystalline phase formed onto the surfaces of the Fe_3_O_4_/SiO_2_ NPs after functionalization with DSBA.

The XRD pattern of the prepared Fe_3_O_4_/SiO_2_-DSBA catalytic system is exhibited in Fig. 6. According to this figure, the peaks that appeared at 2θ = 30.4°, 35.7°, 43.4°, 54.0°, 57.3°, 63.9°, 71.7°, and 74.4°, and are respectively signed by Miller indices of (2 2 0), (3 1 1), (4 0 0), (4 2 2), (5 1 1), (4 4 0), (6 2 0), and (5 3 3), are attributed to the Fe_3_O_4_ magnetic nanoparticles (MNPs) (*JCPDS database: PDF#99–0073*)^62^. The SiO_2_ network gives a broad peak in a range of 2θ = 11.0°–44.0° that overlaps with one of the Fe_3_O_4_ peaks^63^. The other additional peaks that appeared at 2θ = 26.12°, 79.44°, 87.92°, 90.28°, and 95.24° (marked with NP) are related to the new crystalline phase formed on the surface of the Fe_3_O_4_/SiO_2_ MNPs after functionalization with DSBA.

#### Thermogravimetric analysis

**Figure 7:**
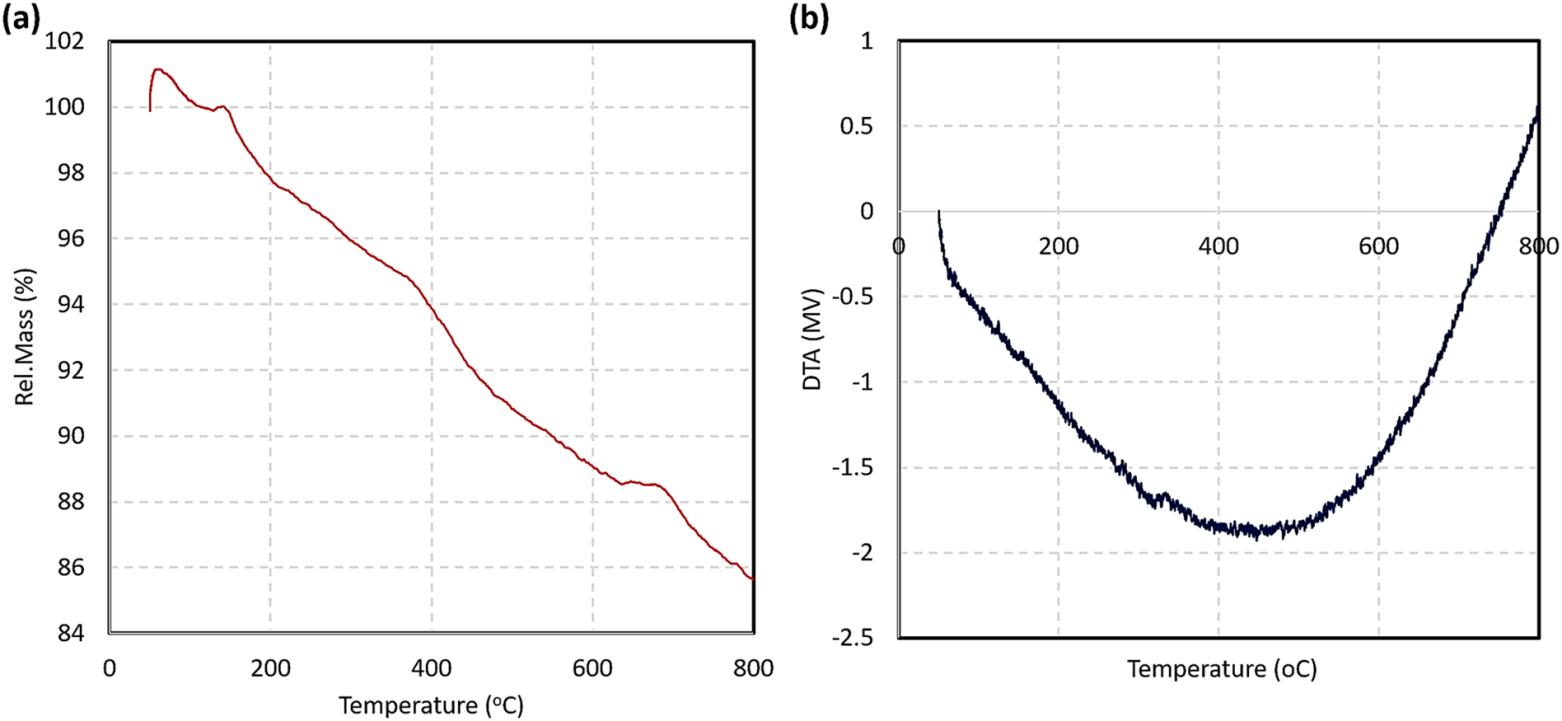
(**a**) TGA curve and (**b**) DTA curve of the designed Fe_3_O_4_/SiO_2_-DSBA catalytic system, under argon atmosphere.

To evaluate thermal stability and decomposition states of the Fe_3_O_4_/SiO_2_-DSBA catalytic system, thermogravimetric analysis (TGA) was performed on the sample under argon atmosphere, in thermal range of 50–800 °C^32^. As presented in Fig. 7a, physical adsorption of the moisture in the air caused a partial increase (1.0%) in the weight, which was quickly returned back by hating the sample up to ca. 120 °C. Then, ca. 5.5% of the total weight was lost by increasing the temperature to around 370 °C, which is attributed to removal of the entrapped water molecules in the silica network^64^. In the next stage, a relatively intense decrease in the weight was occurred through which ca. 6.0% of the total weight was lost. The degradation of the organic structures at this thermal range (300–600 °C) has been confirmed by literature, therefore, this weight loss can be ascribed to decomposition of APS and DSBA organic layer^44^. In continue, a tangible increase in the weight is observed at 630 °C, which may be due to re-adsorption of the combusted materials or adsorption of the argon gas by a porous structure that formed at this temperature^65^. Also, the curve of differential thermal analysis (DTA) was provided for the sample, in the same thermal range. As presented by Fig. 7b, totally an endothermic trend is observed for the Fe_3_O_4_/SiO_2_-DSBA sample, which corroborates well integration and high thermal resistance of the structure. As is seen in the DTA curve, the structure and the used components were not affected by the change in temperature, confirming that functional groups on the surfaces are almost stable.

#### Electron microscopy

**Figure 8:**
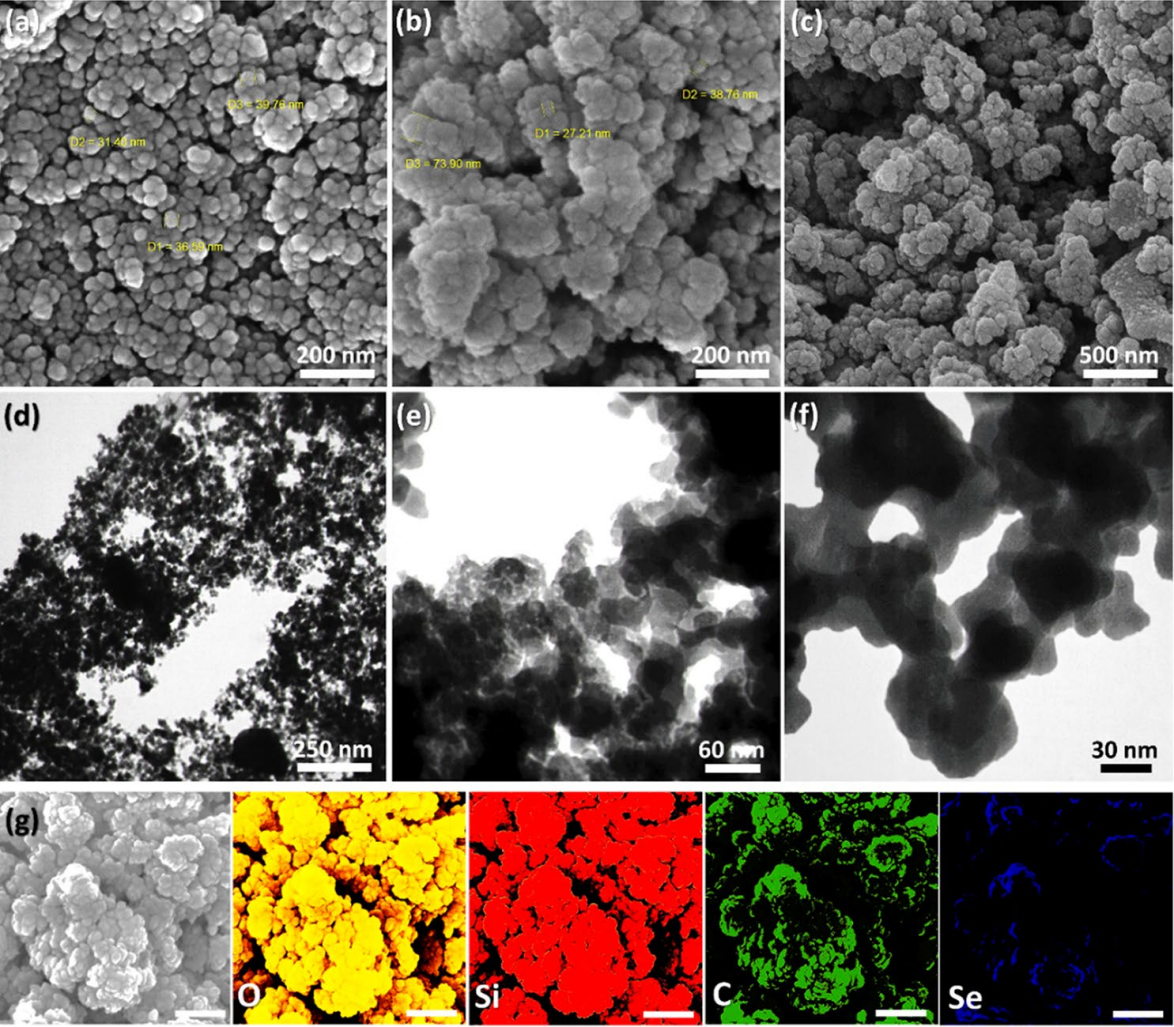
FESEM images of (**a**) Fe_3_O_4_ NPs, (**b**) Fe_3_O_4_/SiO_2_ NPs, (**c**) Fe_3_O_4_/SiO_2_-DSBA, (**d–f**) TEM image of Fe_3_O_4_/SiO_2_-DSBA catalytic system, and (**g**-series) SEM energy-mapping of Fe_3_O_4_/SiO_2_-DSBA catalytic system.

The FESEM and TEM methods were utilized to examine morphology, real structure, size, and dispersion state of the prepared Fe_3_O_4_/SiO_2_-DSBA nanoparticles. As shown in Fig. 8a, Fe_3_O_4_ MNPs have a uniform spherical morphology and are well dispersed, although they are slightly agglomerated after being coated with TEOS, as illustrated in Fig. 8b^53^. According to Fig. 8c, Fe_3_O_4_/SiO_2_-DSBA MNPs are well dispersed and have a spherical morphology. It means that these MNPs provide an extremely active surface area that is required for the catalytic intentions. At this state, the chemical active sites (here *Se-Se* bonds) are quite available to the raw substances. Figure 8d–f confirm successful formation of the core/shell architecture via TEM imaging. In this images, the dark areas are related to the magnetic cores (Fe_3_O_4_ MNPs) and gray areas (light) are related to the shell (SiO_2_-DSBA). The g-series of Fig. 8 is related to the SEM energy-mapping of the prepared Fe_3_O_4_/SiO_2_-DSBA catalytic system, in which each element has been highlighted by a special color. These images better reveal the composition state of the elements and localization of the used ingredients.

#### Mass spectroscopy

The bond energy of dieselnide is only 172 kJ mol^−1^^66^, while this value for C=C, C–H, and C–O are 602, 346, and 358 kJ mol^−1^, respectively. Given these explanations, it is reasonable to expect that the *Se-Se* bond in 2,2′-diselenobis benzoic acid breaks earlier during the mass process, in comparison with the other bonds of this molecular structure. This claim has been proven by the results of mass analysis (MS) on 2,2′-diselenobis benzoic acid sample. The total molecular weight of the symmetric structure of the synthesized 2,2′-diselenobis benzoic acid is 402 g mol^−1^. When this structure undergoes through a mass process, it makes sense that its diselenide bond is broken faster than the rest sites, resulting in the appearance of a signal at 201 g mol^−1^. The mass result of the synthesized 2,2′-diselenobis benzoic acid has been shown in Figure S5 (in SI section), which well confirms breaking of the *Se-Se* bond upon exposure to the excited electrons within the MS analysis.

#### ^1^HNMR and ^13^CNMR analyses on 2,2′-diselenobis benzoic acid compound

**Figure 9:**
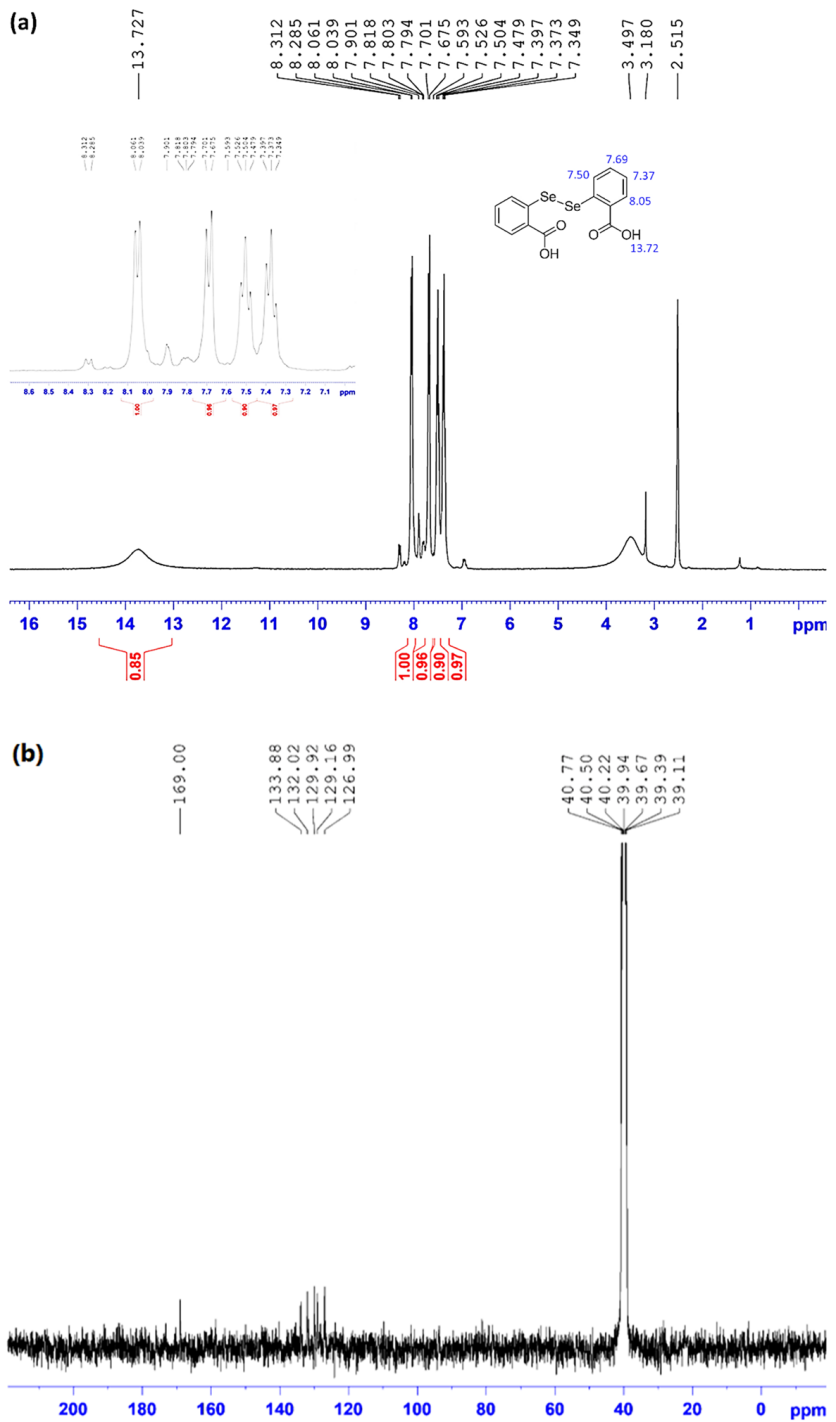
(**a**) ^1^HNMR of 2,2′-diselenobis benzoic acid. (**b**) ^13^CNMR of 2,2′-diselenobis benzoic acid.

For further confirmation of the successful synthesis of 2,2′-diselenobis benzoic acid compound, H- and C-NMR spectroscopy were used. Figure 9 represents the spectral data and the provided NMR spectra that verify successful formation of the synthesized 2,2′-diselenobis benzoic acid structure.

^1^H-NMR (500 MHz, DMSO-d6) δ = 13.73 (bs, 1H, O*H*), 8.05 (d, J = 11.0 Hz, 1H, ArC*H*), 7.69 (d, J = 13.0 Hz, 1H, ArC*H*), 7.50 (t, J = 11 Hz, 1H, ArC*H*), 7.37 ppm (t, J = 12 Hz, 1H, ArC*H*); ^13^C NMR (125.76 MHz, DMSO-d6) = 169.00, 133.88, 132.02, 129.92, 129.16, 126.99 ppm^50^.

### Catalytic application of Fe_3_O_4_@SiO_2_-DSBA in peptide construction

In this section, the catalytic activity of the prepared Fe_3_O_4_@SiO_2_-DSBA system is investigated in the real peptide coupling reactions. To initiate the process, the optimal condition for the amide bond formation between two protected amino acids in the presence of Fe_3_O_4_@SiO_2_-DSBA catalytic system was investigated through examining different factors. In this way, two different methods such as ultrasonication and magnetic stirring have been monitored for the catalytic process. According to literature, ultrasonication can provide a synergistic effect with the heterogeneous particles and positively affect their dispersion state and surface energy of the Fe_3_O_4_@SiO_2_-DSBA particles^67,68^. Hence, this method (abbreviated as US) has also been considered in the experimental stages. Moreover, other effective parameters such as reaction medium, temperature, catalyst amount, and reaction time have been precisely screened. For this purpose, the coupling reaction between glycine methyl ester (Gly-COOMe) and N-protected phenylalanine (Fmoc-Phe-OH) was considered as a model reaction. For further assessments, the same process has been applied for N-protected alanine (Fmoc-Ala-OH), cysteine methyl ester (Cys-COOMe), and N-protected arginine (Fmoc-Arg(pbf)-OH), at the obtained optimal conditions. In continue, the recyclability of the used Fe_3_O_4_/SiO_2_-DSBA catalytic system is experimented and discussed in detail, and a plausible mechanism is suggested for the catalytic process implemented by Fe_3_O_4_/SiO_2_-DSBA system. Finally, a quick comparison is made between the suggested catalytic system in this project and the previously reported ones.

#### Optimization of catalytic values in peptide coupling reactions

In order to determine the optimized conditions for the catalytic process of the Fe_3_O_4_/SiO_2_-DSBA as a coupling reagent for amide bond formation, different experimental conditions including catalyst type and amount, solvent, temperature, time, and the applied method were investigated. For this purpose, the reaction progress was evaluated with thin-layer chromatography (TLC) and ninhydrin spray^32^. As reported in Table 1, the no traceable reaction yield (%) was obtained in the model reaction of the peptide coupling in the absence of the Fe_3_O_4_/SiO_2_-DSBA catalyst, after three hours of stirring in ethanol solvent (Table 1, entry 1). In the same conditions, the reaction yield increased to 38% only by adding 25 mol% of Fe_3_O_4_@SiO_2_ MNPs to the reaction medium (Table 1, entry 2). It means that the Fe_3_O_4_@SiO_2_ particles have provided a suitable substrate for the raw materials to get approach together and start interactions and bonding. It may origin from tight hydrogen-bond interaction between the amino acids and the present hydroxyl groups onto the surface of the particles. As is observed in Table 1(entry 3), this value reached to 89% through subjection of Fe_3_O_4_/SiO_2_-DSBA catalyst to the reaction, at the same conditions. Obviously, this difference comes from DSBA and its related interactions with the raw materials. Further, the effects of the reaction time, amount of catalyst, and reaction medium were precisely monitored. From the perfumed control experiments, it was disclosed that the highest yield is obtained via using 25 mol% of Fe_3_O_4_/SiO_2_-DSBA particles in ethanol over 180 min. Also, from a comparison between the applied methods, it was revealed that the stirring better works than the ultrasonication. Although, a better dispersion state is obtained for the catalyst’s particles under the ultrasonication conditions, it seems that the Se-Se site is not stable enough to tolerate the ultrasound waves. The water medium and even solvent-free conditions were experimented for the catalytic process. As is observed in Table 1(entries 14 and 15), very low reaction yields were obtained at the mentioned conditions. For the water medium, it may originate from inappropriate dispersion of the particles due to the presence of the propyl groups (as a hydrophobic agent) on the surfaces^69^. For the solvent-free conditions, a ball-milling equipment was used, and it was found out that the Fe_3_O_4_/SiO_2_-DSBA structure is sensitive to mechanical hitting, and is damaged. As well, it was mentioned in characterization section (MS analysis) that the Se-Se bond is sensitive to the excited electrons, and quickly breaks down. The determined optimal condition was applied in some additional peptide coupling reactions, and the obtained dipeptide structures were recognized with H-NMR spectroscopy, as presented in Figures S6–S8 (in the SI section). According to the above descriptions, other dipeptide structures (reported in the SI section) were synthesized under the optimal catalytic conditions.

**Table 1:** Optimization of the amidation reaction between glycine methyl ester and Fmoc-protected phenylalanine, catalyzed by Fe_3_O_4_/SiO_2_-DSBA nano-system.


Entry	Conditions	Solvent	Time (min)	C^a^ (mol%)	Yield^b^ (%)
1	Catalyst-free/stirring/r.t	EtOH	180	–	N.R
2	Fe_3_O_4_@SiO_2_ NPs/stirring/r.t	EtOH	180	0.25	38
3	Fe_3_O_4_/SiO_2_-DSBA NPs/stirring/r.t	EtOH	180	0.25	89*
4	Fe_3_O_4_/SiO_2_-DSBA NPs/stirring/r.t	EtOH	90	0.25	64
5	Fe_3_O_4_/SiO_2_-DSBA NPs/stirring/r.t	EtOH	210	0.25	87
6	Fe_3_O_4_/SiO_2_-DSBA NPs/stirring/r.t	EtOH	180	0.12	73
7	Fe_3_O_4_/SiO_2_-DSBA NPs/stirring/r.t	EtOH	180	0.37	90
8	Fe_3_O_4_/SiO_2_-DSBA NPs/US/r.t	EtOH	30	0.25	48
9	Fe_3_O_4_/SiO_2_-DSBA NPs/US/r.t	EtOH	60	0.25	40
10	Fe_3_O_4_/SiO_2_-DSBA NPs/stirring/40	EtOH	180	0.25	82
11	Fe_3_O_4_/SiO_2_-DSBA NPs/stirring/55	EtOH	180	0.25	76
12	Fe_3_O_4_/SiO_2_-DSBA NPs/stirring/55	DMF	180	0.25	78
13	Fe_3_O_4_/SiO_2_-DSBA NPs/stirring/55	DCM	180	0.25	74
14	Fe_3_O_4_/SiO_2_-DSBA NPs/stirring/r.t	H_2_O	180	0.25	55
15	Fe_3_O_4_/SiO_2_-DSBA NPs/ball-milling	SF^c^	30	0.25	Trace

^a^C stands for catalyst ratio; ^b^Isolated yield; r.t. stands for room temperature; US stands for ultrasonication; Reaction conditions: 4.0 mmol of the first amino acid (N-protected), 4.6 mmol of acid-protected amino acid, 10.0 mL of ethanol, and 0.25 mol% of Fe_3_O_4_/SiO_2_-DSBA catalytic system; *Optimum conditions; ^c^ SF: solvent free; The turnover number (TON) and turnover frequency (TOF) values were estimated to be 356 and 3.3 × 10^−2^ (s^−1^), respectively (given in the SI section).

#### Recyclability of Fe_3_O_4_/SiO_2_-DSBA catalytic system

**Figure 10:**
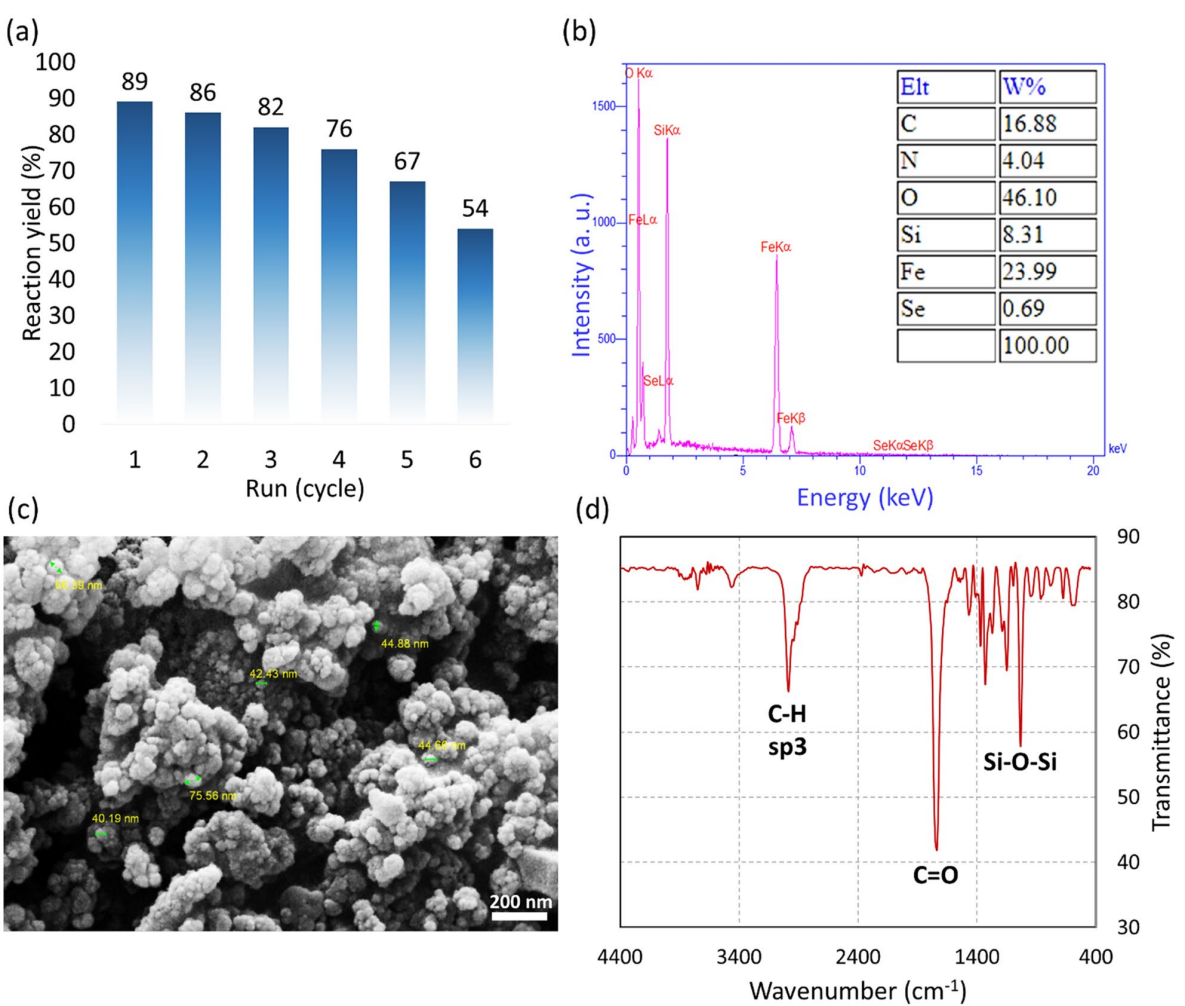
(**a**) Recyclability investigation of Fe_3_O_4_/SiO_2_-DSBA nanoparticles in catalyzed peptide coupling reactions. The results were obtained from the coupling reaction between glycine methyl ester and Fmoc-protected phenyl alanine, per 0.25 mol% of the catalyst at room temperature, (**b**) EDX data, (**c**) SEM image, and (**d**) FTIR spectrum of the recovered Fe_3_O_4_/SiO_2_-DSBA nanoparticles after six times recycles.

The reusability of the prepared Fe_3_O_4_/SiO_2_-DSBA catalytic system in amid bond formation was evaluated in the model reaction of glycine methyl ester and protected phenylalanine. For this aim, after completion of the reaction, the Fe_3_O_4_/SiO_2_-DSBA nanoparticles were separated from the reaction mixture by an external magnet and then washed with distilled water, and then dried in an oven in order to get ready for the next catalytic run. Then, the recovered catalyst in a constant amount was utilized for additional five subsequent runs. According to Fig. 10a, a partial reduction (7%) in catalytic performance of the recovered Fe_3_O_4_/SiO_2_-DSBA was observed, but a sharp decrease was occurred during the next recycles until 35% of the initial value was lost. As the most probable contributor to this, it can be stated that there was a severe agglomeration in the recovered particles after the third and fourth runs. At the first stages of recyclization, irradiation of the ultrasound waves (50 kHz, 100 W L^−1^) led to well re-dispersion of the particles, but severe agglomeration after the third run reduced the total performance of the catalyst. The mentioned agglomeration that is occurred due to the paramagnetic behavior of the Fe_3_O_4_/SiO_2_-DSBA particles, causes the active catalytic sites (Se-Se) to be blocked and significantly reduced^54^. Therefore, the catalytic performance is sharply dropped after several times utilization and recovery, and longer times of ultrasonication is needed. According to literature, long time ultrasonication in the cleaner bath can cause damage to the core/shell structure of Fe_3_O_4_/SiO_2_^69^. Figure 10b, c show the results of the EDX and SEM analyses on the recovered Fe_3_O_4_/SiO_2_-DSBA nanoparticles after six successive usages. According to Fig. 10b, after six consecutive uses, the Fe_3_O_4_/SiO_2_-DSBA nanocatalyst still has the main element of its catalytic site, (selenium), which can be a reason for a yield of 54% after six consecutive uses. According to Fig. 10c, the morphology, uniformity, and size of the Fe_3_O_4_/SiO_2_-DSBA catalytic system have not significantly changed compared to the first use, but particles agglomeration is clearly confirmed by the prepared SEM image. Also, Fig. 10d reveals that no changes in the present functional groups onto the surface of the Fe_3_O_4_/SiO_2_-DSBA particles occurred during the recyclization, as the sharp peaks related to the stretching vibrations of Si‒O‒Si, C=O, and C‒H bonds are still seen in the prepared FTIR spectrum. Based on these results, this is concluded that the presented Fe_3_O_4_/SiO_2_-DSBA catalytic system includes economic benefits in comparison with the homogeneous analogues, as they are not able to be recycled and reused for several times.

#### Suggested mechanism

**Figure 11:**
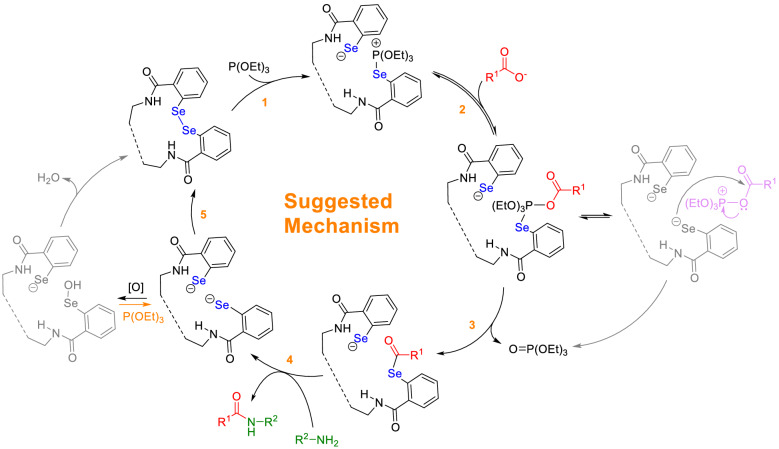
A plausible mechanism suggested for the catalyzed amidation reaction by Fe_3_O_4_/SiO_2_-DSBA catalytic system.

A plausible mechanism for the catalyzed amide/peptide bond formation by the prepared Fe_3_O_4_/SiO_2_-DSBA system is schematically presented in Fig. 11^48^. As is observed, totally five stages should be passed to achieve the intended amide/peptide bond and the recovered Fe_3_O_4_/SiO_2_-DSBA. The first stage of this mechanism begins by insertion of triethyl phosphite as an initial reducing agent^70^. At this stage, the attachment of phosphorus atom to one of the involved selenium atoms creates a phosphonium structure which is an active intermediate. In stage 2, the carboxylate group in the structure of the first amino acid is attached to the phosphonium center. In the third stage, a selenide attacks to the carbonyl group, and a triethyl phosphate (O = P(OEt)_3_) is subsequently released^71^. At this state, the first amino acid is active and ready for the attachment of the amine group from second amino acid. The next stage involves the attack of the amine group of the second amino acid to the carbonyl group of the first amino acid leading to the formation of a peptide bond. In the final stage (stage 5), the negatively charged selenium is oxidized by the oxygen in the air^72^, and the initial structure of DSBA is recovered through elimination of a water molecule.

#### Comparisons

So far, several heterogeneous catalytic systems have been suggested for facilitating the amide/peptide bond formation, because this type of chemical couplings is of high importance in the current pharmaceutical researches^73^. Hence, it would be essential to highlight the advantageous of these catalytic systems for further consideration by the researchers in the field. As discussed in the introduction section, high heterogeneity and paramagnetic behavior of the designed Fe_3_O_4_/SiO_2_-DSBA catalytic system can be mentioned as the foremost merits that provide this great opportunity to conveniently separate and recover the particles for successive utilization. Therefore, in comparison with the homogenous species (Table 2, entries 1–4), the proposed Fe_3_O_4_/SiO_2_-DSBA catalytic system includes merit for utilization and recyclization. Form economic aspect, it was clearly presented in this report that inexpensive materials were used that are quite available in the laboratories. So, preparation of the presented catalytic system would be reasonable for large-scale utilization. In comparison with the similar systems that include magnetic property (Table 2, entry 5), exploitation of diselenide compounds are safer than the isothiazolone (IT) derivatives, which can cause severe side effects such as skin irritations and allergies^74^. As well, the used amount of the catalyst particles is less in the case of Fe_3_O_4_/SiO_2_-DSBA system, confirming higher efficiency than the other similar systems. Table 2 provides information on several catalysts that are capable of catalyzing the formation of amide bonds. This table can be used to compare the performance of the Fe_3_O_4_/SiO_2_-DSBA catalytic system with the other catalysts with a quick glance. Given the yield percentage and reaction condition of the method presented in this study, it seems that this method deserves much attention.

**Table 2:** Comparison of the catalytic performance of the Fe_3_O_4_/SiO_2_-DSBA with some other catalysts for the amidation reaction.

Entry	Catalyst	Product	Catalyst loading	Conditions	Yield^a^ (%)	Refs.
1	Diboronic acid anhydride (DBAA)	Boc-Ser-Gly-OBn	2.0 μmol, 2.0 mol %	DCE, 90 °C, 4 h	88	^75^
2	(2-(Thiophen-2-ylmethyl)phenyl)boronic acid	(S,S)-N-Boc-Phe-Val methyl ester	25 mol %	PhF, 65 °C, 24 h	50	^76^
3	Polymer-supported BOP (P-BOP)^a^	BocAib-PheOEt	0.30 g	DCM, r.t., 18 h	75	^77^
4	Ta(OMe)_5_	Boc-L-Cys(t-Bu)–L-Ala-Ot-Bu	33.6 mg, 0.10 mmol	60 °C, 72 h	97	^78^
5	Ag/Fe_3_O_4_@SiO_2_-IT^b^	Fmoc-Ala-Gly-OMe	0.2 g	Dry DCM, r.t., 4 h	92	^32^
6	Fe_3_O_4_/SiO_2_-DSBA	Fmoc-Phe-Gly-OMe	0.1 g, 0.25 mol%	Ethanol, r.t., 3 h	89	This work

^a^BOP stands for benzotriazol-1-yloxytris(dimethylamino)phosphonium hexafluorophosphate; ^b^IT stands for isothiazolone.

## Experimental section

### Materials and equipment

All the chemicals, reagents, and equipment used in this study are listed in Table 3.

**Table 3:** Chemicals and equipment used in this study.

Materials and equipment	Purity and brand
FeCl_2_·4H_2_O	Sigma Aldrich (98%)
FeCl_3_·6H_2_O	Sigma Aldrich (≥ 98%)
Ammonia	Merck (25%)
Ethanol	Sigma Aldrich (97%)
Tetraethyl orthosilicate	Sigma Aldrich (98%)
(3-Aminopropyl)triethoxysilane	Sigma Aldrich (99%)
Polyethylene glycol 300	Sigma Aldrich
Toluene	Merck (99.8%)
Selenium	Merck (99.99%)
Potassium hydroxide	Merck (≥ 85%)
potassium tetrahydroborate	Merck (98%)
Anthranilic acid	Sigma Aldrich (98%)
Hydrochloric acid	Sigma Aldrich (37%)
Sodium nitrite	Merck (≥ 97%)
Methanol	Merck (99.8%)
Dimethyl sulfoxide	Merck (99.9%)
DIEA	Merck (95%)
TBTU	Merck (97%)
Fmoc-Phe-OH	Sigma-Aldrich
Fmoc-Cys (Trt)-OH	AChem Block Co
Fmoc-Ala-OH	Sigma-Aldrich
H-Gly-OMe HCl	Sigma-Aldrich
Fmoc-Arg(Pbf)-OH	AChem Block Co
Dichloromethane	Sigma-Aldrich, ≥ 99.0%
Piperidine	Sigma-Aldrich, ≥ 99.0%
Trifluoroacetic acid (TFA)	Sigma-Aldrich, ≥ 99.0%
Diethyl ether	Merck,98%
Ethyl acetate	Merck, 98%
Magnesium Sulphate	Sigma-Aldrich, ≥ 95.0%
Ninhydrin	Sigma Aldrich
FT-IR analysis	Shimadzu IR-470 spectrometer
EDX analysis	Numerix DXP–X10P
H-NMR 500 MHz	INOVA 5
TGA	TG 209F3 NETZSCH
C-NMR 500 MHz	INOVA 5
Mass Spectrometer	5975C
FE-SEM analysis	Sigma-Zeiss, microscope
VSM analysis	Lakeshore 7407
XRD analysis	JEOL JDX–8030 (30 kV, 20 mA)
Ultrasound cleaning bath	KQ-250 DE (40 kHz, 250 W)
Rotary evaporator	Heidolf
Vacuum oven	DZF-6020-HT-400P

### Preparation methods

#### Preparation of K_2_Se_2_

Initially, 4.38 mmol of selenium element powder was transferred into a round-bottom flask (50 mL), and the reflux system was set up at room temperature, under N_2_ atmosphere. Then, 6.6 mmol of KOH and 0.55 mmol of KBH_4_ were poured into a beaker which was in the ice bath, and then 4.0 mL of deionized water was added and then stirred with a glass stirrer to obtain a clear solution. The resulting clear solution was added into the selenium-containing flask using a syringe. Next, the content of the flask was stirred vigorously under reflux conditions (90 °C) for an hour to obtain a red–black solution.

#### Synthesis of DSBA

For the synthesis of 2-carboxybenzenediazonium chloride, in a round-bottom flask (50 mL), anthranilic acid (4.38 mmol) was dissolved in deionized water (8.0 mL) via stirring. Then, 0.5 mL of HCl was added into the flask through a dropwise manner to obtain a clear solution. After complete dissolution, the flask was transferred into an ice bath including salt and acetone (0 °C). Then, NaNO_2_ (5.27 mmol) was dissolved in 1.5 mL of deionized water in a separate beaker. The NaNO_2_ solution was then added dropwise into the anthranilic acid-containing flask, which had been placed in an ice bath. Next, the resulting solution was stirred at 0 °C for 45 min. In the next step, the metal salt solution of K_2_Se_2_, which was synthesized in the previous step, was added dropwise to the solution in the ice bath. The flask was then cooled down to room temperature. Afterward, it was stirred vigorously at 90 °C for 2 h until a dark red solution was precipitated at the end of the reaction flask. Again, the flask was cooled down to room temperature. Next, to separate the unreacted and oxidized selenium from the products, the resulting solution was filtered by a thin celite pad. The presence of a small amount of the unreacted selenium on the celite pad indicated that the majority of the primary material has been converted to Se^2−^ form. In the last step, HCL (7.0 mL, 1.0 M) was added to the filtrate and then the resulting precipitates were filtered through a paper filter. The resulting precipitate was recrystallized with hot methanol for purification^79^.

#### Preparation of Fe_3_O_4_ nanoparticles

In a three-necked round-bottom flask (500 mL), 20.0 mmol of FeCl_3_·6H_2_O and 20.0 mmol of FeCl_2_·4H_2_O were dissolved in 200.0 mL of deionized water, via ultrasonication for 20 min. The flask was then placed in an oil bath and heated up to 45 °C, and stirred under N_2_ atmosphere. After complete dissolution, the temperature was slowly raised to 85 °C and the mixture was vigorously stirred at the same condition for additional 2 h. Then, 30.0 mL of concentrated ammonia solution (25%) was dropwise added into the mixture for 1 h. Finally, after cooling the solution, the magnetic nanoparticles were collected by an external magnet and washed several times with deionized water, ethanol and acetone, and dried in oven (60 °C).

#### Preparation of Fe_3_O_4_@SiO_2_ nanoparticles

In a round-bottom flask (50 mL), 1.0 g of Fe_3_O_4_ NPs was placed and 10.0 mL of deionized water, 5.0 mL of ethanol, 5.0 mL of PEG-300, and 1.0 mL of ammonia were added and stirred at room temperature. Next, 2.0 mL of tetraethylorthosilicate (TEOS) was dissolved in 10.0 mL of ethanol in a separate flask, and the resulted solution was dropwise added into the flask containing Fe_3_O_4_ NPs, and the content was stirred for 12 h at room temperature. In the next step, the obtained Fe_3_O_4_@SiO_2_ nanoparticles were collected by an external magnet and washed several times with deionized water as well as ethanol, and ultimately dried in oven (60 °C).

#### Preparation of Fe_3_O_4_@SiO_2_-NH_2_ nanoparticles

In a round-bottom flask (100 mL), 2.0 g of Fe_3_O_4_@SiO_2_ that was prepared in the previous stage, was dispersed in toluene (50.0 mL) via ultrasonication for 15 min. Then, 4.0 mL of 3-aminopropyltriethoxysilane (APTES) was added into the flask, and the reflux was set up at 110 °C under N_2_ atmosphere. The content was vigorously stirred for 12 h. Finally, after cooling down to room temperature, the obtained Fe_3_O_4_@SiO_2_-NH_2_ nanoparticles were collected by an external magnet and washed several times with toluene.

#### Preparation of Fe_3_O_4_/SiO_2_-DSBA catalytic system

In a round-bottom flask (25 mL), 0.7 g of Fe_3_O_4_@SiO_2_-NH_2_ was dispersed in 3.0 mL of dimethylsulfoxide (DMSO), under N_2_ atmosphere. Then, in a separate flask, 0.1 g of the synthesized DSBA was dissolved in 3.0 mL of DMSO, and in another flask, 0.2 mL of diisopropylethylamine (DIEA) was mixed with 2.0 mL of DMSO and 0.095 g of TBTU. Then, these two solutions were simultaneously added into the main flask containing Fe_3_O_4_@SiO_2_-NH_2_ using a syringe (drop-by-drop), and stirred at room temperature for 2 h. Finally, the Fe_3_O_4_/SiO_2_-DSBA nanoparticles were collected by an external magnet and washed several times with water and ethanol, and dried at room temperature.

### General procedure of amide/peptide bond formation catalyzed by Fe_3_O_4_/SiO_2_-DSBA system

In a round-bottom flask (25 mL), Fe_3_O_4_/SiO_2_-DSBA particles (0.1 g, 0.25 mol%) were dispersed in ethanol (10.0 mL) using an ultrasound bath (50 kHz, 100 W L^−1^), and P(OEt)_3_ (0.1 mol) was added into the mixture. Next, 4.0 mmol of the first amino acid (N-protected) was added into the flask, and the resulting mixture was stirred for 30 min at room temperature. Then, 4.6 mmol of acid-protected amino acid was added and the resulting mixture was stirred at room temperature, for 3 h under air atmosphere. After this time, the nanoparticles of Fe_3_O_4_/SiO_2_-DSBA were separated from the reaction medium by an external magnet and washed several times with ethanol and then dried in an oven at 60 °C to be reused if necessary. For the purification of the synthesized dipeptide compound, 10.0 mL dichloromethane (DCM) and 5.0 mL of deionized water were added to the solution, and the mixture was transferred into a separatory funnel (100 mL) and well mixed. The phosphate compound and unreacted amino acid (acid-protected) are removed via separation of DCM from aqueous phase. Then, the DCM phase was dehydrated through addition of magnesium sulfate powder (0.5 g). After 30 min, the swollen magnesium sulfate crystals were separated via paper filtration, and the remained solution was dried by rotary evaporator. To have a clean NMR spectrum of the synthesized Cys-Arg dipeptide structure, removal of the protecting groups was essential to be performed. For this purpose, 2.0 mmol of the obtained Cys-Arg (protected) was dissolved in DCM (4.0 mL), and then piperidine (2.0 mL, 0.25% in DCM) was added into the solution, and stirred for 30 min at room temperature. Then, the flask was put into an ice bath and cold diethyl ether was gradually added to the solution during gentle stirring. The obtained white powder was separated via filtration with a sintered glass filter, and dried in the vacuum oven. The powder was then dissolved in ethyl acetate (2.0 mL) and trifluoroacetic acid (8.0 mL, 95% in water) and stirred for 30 min, at 10 °C in an ice bath. Finally, the solution was concentrated by rotary evaporator, and cold diethyl ether was gradually added to the solution during gentle stirring until the color of the solution turned into white. The obtained white powder was separated via filtration with a sintered glass filter, and dried in the vacuum oven.

## Conclusion

In continuing our previous efforts in preparation of the heterogeneous peptide coupling reagents, a nanoscale catalytic system has been designed and successfully applied in rapid formation of the amide/peptide bond between the amino acids in the solution phase. In this regard, a simple core/shell structure of Fe_3_O_4_/SiO_2_ nanoparticles has been constructed and functionalized with 2,2′-diselenobis(benzoic acid) (DSBA), as the main catalytic site for amide/peptide bonding. The DSBA structure has been synthesized through organic synthesis techniques, and then identified by NMR and MS spectroscopic methods. After full characterization of the catalyst’s structure, its capability in assisting the amide bond formation was investigated in the solution-phase dipeptide constructions, wherein ca. 89% reaction yield was obtained at optimal conditions (180 min, room temperature). The protected amino acids including Fmoc-Ala-OH, Fmoc-Phe-OH, Fmoc-Arg(pbf)-OH, and glycine methyl ester were purchased and experimented to screen the catalytic process. In this account, a plausible mechanism has been suggested for the catalytic process in which sensitive role of the diselenide bond was highlighted, based on the supportive resources. Concisely, a red/ox process is driven by triethyl phosphine through which the diselenide bond in the structure of DSBA is opened, and the carboxylate group of the amino acid is activated. The structure of DSBA is then recovered through oxidation by the air. Due to showing a substantial paramagnetic behavior, the Fe_3_O_4_/SiO_2_-DSBA particles were conveniently collected, revived, and reused in three successive catalytic runs with only 7% reduction in the total performance. Overall, due to all mentioned brilliant points for the presented nanocatalyst, large-scale fabrication and utilization is the industrial applications is recommended. As a point that may be focused in future practices, the preparation method of the proposed catalyst can be modified, since the active diselenide bond may be affected to some extent within the covalent attachment onto the particles surfaces. Hence, it can be a challenging suggestion for the next efforts in the same field of research.

## Supplementary Information

Supplementary Information 1.

Supplementary Video 1.

## Data Availability

All data generated or analyzed during this study are included in this published article and its supplementary information file.
