# Supplementary material for: A diselenobis-functionalized magnetic catalyst based on iron oxide/silica nanoparticles suggested for amidation reactions
Source: Sci Rep. 2022 Sep 1;12:14865. doi: 10.1038/s41598-022-19030-w (PMC9436994; doi:10.1038/s41598-022-19030-w)
Supplement: Supplementary file 1 — Supplementary Information 1. [file 41598_2022_19030_MOESM1_ESM.docx]

**Supporting Information**

**A Diselenobis-Functionalized Magnetic Catalyst Based on Iron Oxide/Silica Nanoparticles Suggested for Amidation Reactions**

Reza Taheri-Ledari, Fateme Sadat Qazi, Mahdi Saeidirad, Ali Maleki*

*Catalysts and Organic Synthesis Research Laboratory, Department of Chemistry, Iran University of Science and Technology, Tehran 16846-13114, Iran.*

**Corresponding author. E-mail:* [*maleki@iust.ac.ir*](mailto:maleki@iust.ac.ir)*; Fax: +98-21-73021584; Tel: +98-21-73228313.*


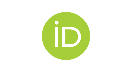
*Author’s ORCIDs:*

*Reza Taheri-Ledari:* [*https://orcid.org/0000-0002-6511-9411*](https://orcid.org/0000-0002-6511-9411)

*Ali Maleki:* [*https://orcid.org/0000-0001-5490-3350*](https://orcid.org/0000-0001-5490-3350)

***Table of Content***

| Content | Page |
| --- | --- |
| **Figure S1**-The FTIR spectrum of Fe_3_O_4_ | **S2** |
| **Figure S2**-The FTIR spectrum of Fe_3_O_4_@SiO_2_ | **S3** |
| **Figure S3**-The FTIR spectrum of Fe_3_O_4_@SiO_2_-NH_2_ | **S4** |
| **Figure S4**-The FTIR spectra of Fe_3_O_4_/SiO_2_-DSBA | **S5** |
| **Figure S5**-The Mass result of 2,2’-diselenobis benzoic acid | **S6** |
| **Figure S6**. H-NMR Spectra and spectral data of Fmoc-Ala-Gly-OMe dipeptide structure | **S8** |
| **Figure S7**. H-NMR Spectra and spectral data of Fmoc-Phe-Gly-OMe dipeptide structure | **S9** |
| **Figure S8**. 1H NMR spectrum for compound Cys-Arg dipeptide structure | **S10** |
| **TON and TOF values** | **S11** |
| **Supplementary Video 1:** Diselenobis Recrystallization. | **S11** |

***Figure S1****. The FTIR spectrum of Fe_3_O_4_ NPs.*

***Figure S2****. The FTIR spectrum of Fe_3_O_4_@SiO_2_ NPs.*

***Figure S3.*** *The FTIR spectrum of Fe_3_O_4_@SiO_2_-NH_2_.*

***Figure S4.*** *The FTIR spectrum of Fe_3_O_4_/SiO_2_-DSBA.*


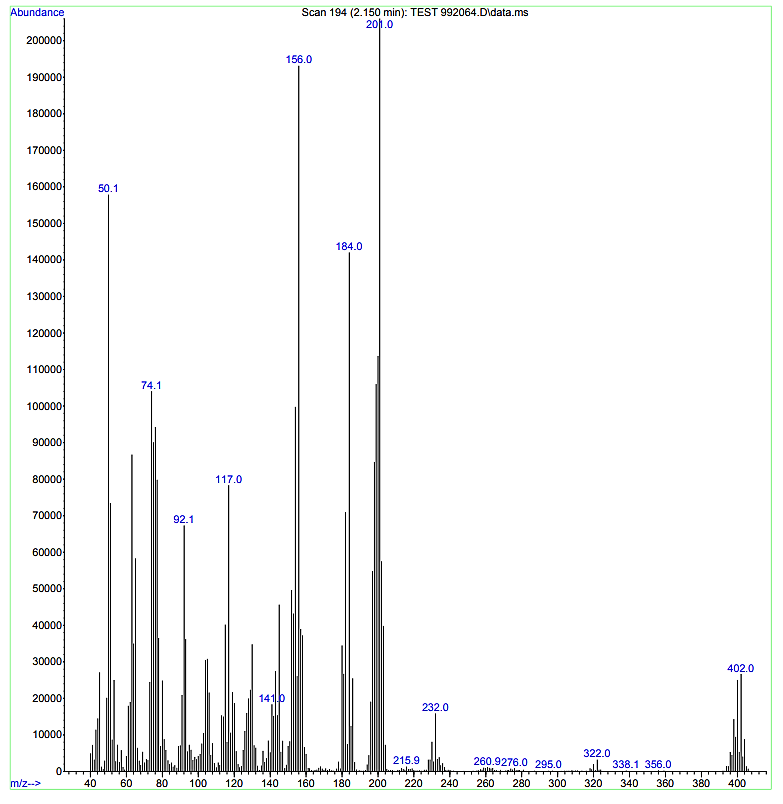


***Figure S5.*** *The Mass spectroscopy result of 2,2’-diselenobis benzoic acid.*

C_14_H_10_O_4_Se_2_

M: 401 g/mol

[M+1]^+^ , *m/z* = 402

C_7_H_6_O_2_Se

M: 201.95 g/mol

*m/z* = 201

**H-NMR Spectra and spectral data**

***Fmoc-Ala-Gly-OMe***

^1^H NMR (500 MHz, DMSO): δ = 1.26 (d, 3H, J = 7 Hz, CHC*H_3_*), 3.64 (s, 3H, COOC*H_3_*), 3.80-3.95 (qd, 1H, J = 16.5 Hz, J = 6.5 Hz, NHC*H*CH_3_), 4.12 (t, 1H, J = 6.5 Hz, C*H*CH_2_O), 4.23 (d, 2H, J = 6.5 Hz, CHC*H_2_*O), 4.27 (s, 2H, NHC*H_2_*CO), 7.34 (t, 2H, J = 7 Hz, Ar), 7.43 (t, 2H, J = 7 Hz, Ar), 7.58 (d, 1H, J = 7.5 Hz, OCON*H*CH), 7.73-7.76 (t, 2H, J = 7.5 Hz, Ar), 7.90 (d, 2H, J = 7.5 Hz, Ar), 8.31 (s, 1H, CON*H*CH_2_).


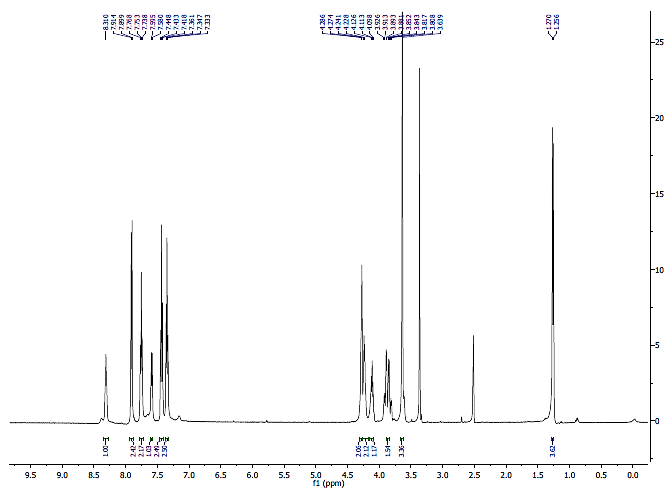


**Figure S6**. H-NMR Spectra and spectral data of Fmoc-Ala-Gly-OMe dipeptide structure.

***Fmoc-Phe-Gly-OMe***

^1^H NMR (500 MHz, DMSO): δ = 2.81 (t, 1H, J = 11 Hz, PhC*H_2_*), 3.06 (d, 1H, J = 15 Hz, PhC*H_2_*), 3.65 (s, 3H, COOC*H_3_*), 3.89-3.93 (m, 2H, NHC*H_2_*COOCH_3_), 4.10-4.18 (m, 3H, C*H*C*H_2_*OCONH), 4.29-4.34 (td, 1H, J = 3.5 Hz, J = 12.5 Hz, NHC*H*CH2Ph), 7.18-7.2 (t, 1H, J = 5, CON*H*CH_2_COOCH_3_), 7.25-7.35 (m, 7H, Ar), 7.39-7.43 (m, 2H, Ar), 7.63-7.71 (m, 2H, Ar), 7.88 (d, 2H, J = 7.5 Hz, Ar), 8.54 (d, 1H, J = 5.5 Hz, OCON*H*CH).

**
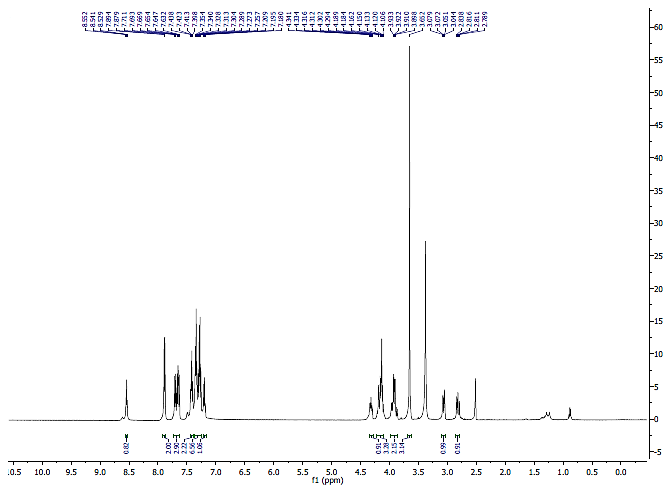
**

**Figure S7**. H-NMR Spectra and spectral data of Fmoc-Phe-Gly-OMe dipeptide structure.

**Cys-Arg**

^1^H NMR (500 MHz, DMSO): δ = 1.58-1.66 (m, 2H, C*H2*), 1.73-1.83 (m, 2H, C*H2*), 2.02 (t, 2H, *J*= 10 Hz, C*H2*), 2.79 (s, 1H, SH), 3.01-3.17 (m, 2H, CH2), 3.86 (t, 1H, J = 10 Hz, CH), 5.10 (t, 1H, *J*= 10.5 Hz, CH), 6.28 (s, 4H, NH_2_), 7.71 (s, 2H, NH_2_), 8.06 (s, 1H, N*H*).


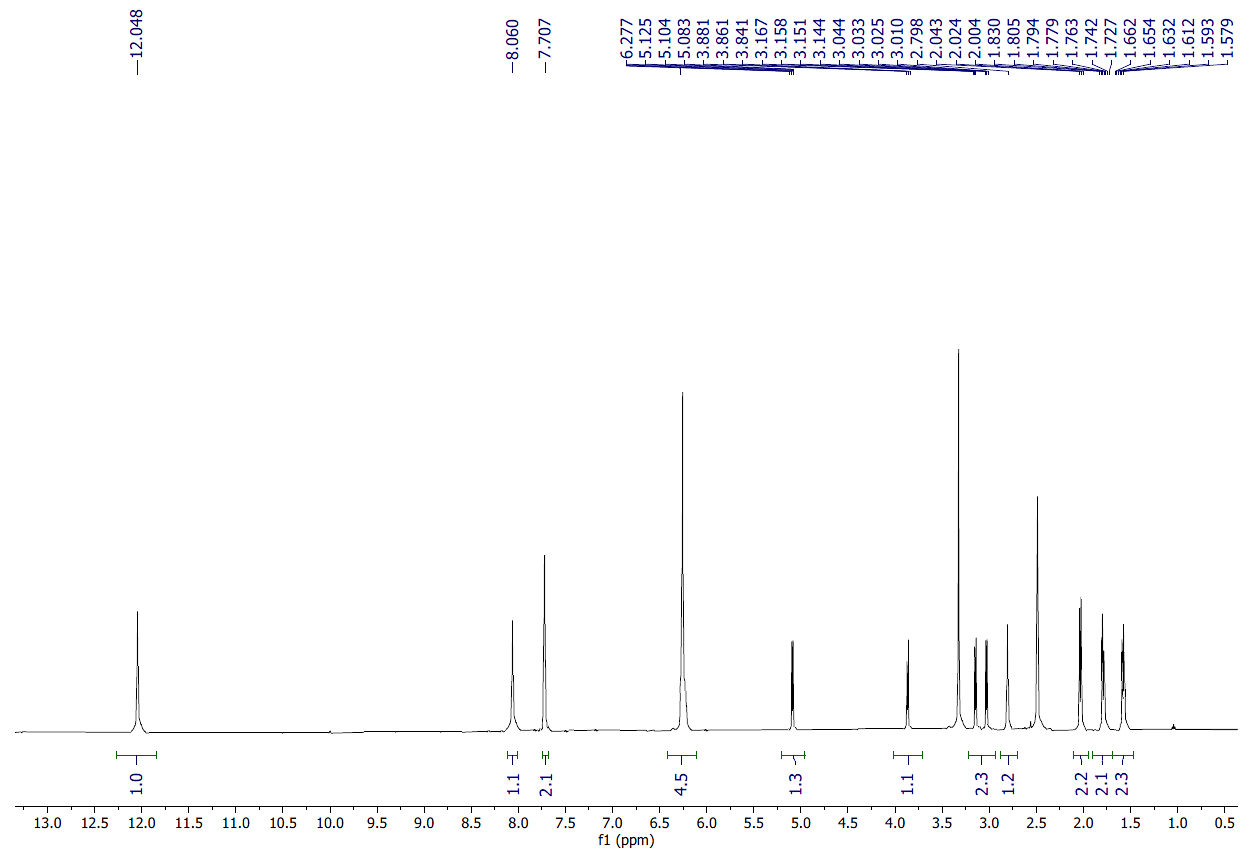


**Figure S8**. ^1^H NMR spectrum for compound Cys-Arg.

**TON and TOF values**

In optimum conditions (Table 1, entry 3), the turnover number (TON) and turnover frequency (TOF) can be estimated as below;

TON = number of moles of reactant consumed/mole of catalyst = 89.0 mol% / 0.25 mol% = 356

Turnover frequency (TOF) = TON/time of reaction = 356/10800 (s) = 3.3 × 10^-2^ (s^-1^)

**Supplementary Video 1:** Diselenobis Recrystallization.

The recrystalization of diselenobis compound can be found as mp4 file via the journal’s online website.
